# Ursodeoxycholic acid prompts glycolytic dominance, reductive stress and epithelial-to-mesenchymal transition in ovarian cancer cells through NRF2 activation

**DOI:** 10.1038/s41420-025-02398-9

**Published:** 2025-04-03

**Authors:** Adrienn Sipos, Éva Kerekes, Dóra Szeőcs, Fanni Szarvas, Szandra Schwarcz, Emese Tóth, Gyula Ujlaki, Edit Mikó, Peter Bai

**Affiliations:** 1 Department of Medical Chemistry, Faculty of Medicine, University of Debrecen, Debrecen, Hungary; 2 HUN-REN Cell Biology and Signaling Research Group, Debrecen, Hungary; 3 The Hungarian Academy of Sciences, Center of Excellence, Debrecen, Hungary; 4 MTA-DE Lendület Laboratory of Cellular Metabolism, Debrecen, Hungary; 5 Research Center for Molecular Medicine, Faculty of Medicine, University of Debrecen, Debrecen, Hungary

**Keywords:** Cancer metabolism, Ovarian cancer, Mechanisms of disease

## Abstract

Numerous secreted bacterial metabolites were identified with bioactivity in various neoplasias, including ovarian cancer. One such metabolite is ursodeoxycholic acid (UDCA), a secondary bile acid that has widespread beneficial effects in neoplasias. Hereby, we assessed the bioactivity of UDCA in cell models of ovarian cancer, by applying UDCA in concentrations corresponding to the serum reference concentrations of UDCA (300 nM). UDCA induced epithelial-to-mesenchymal transition (EMT), increased the flux of glycolysis and reduced the naturally occurring oxidative stress in ovarian cancer cells. These changes were dependent on the activation of NRF2. The tumoral overexpression of UDCA-induced genes in humans correlated with worse survival. These results point out that bacterial metabolites may have opposite effects in different neoplasias and raise the possibility that UDCA-containing remedies on the long run may support cancer progression in ovarian cancer patients.

## Introduction

Ovarian cancer is the second most common and most lethal gynecological malignancy after endometrial cancer [1, 2]. Ovarian cancer is not a single homogenous disease, but can be classified into two major types, type I and II with differences in the mechanism of carcinogenesis, tissue of origin and clinical prognosis [3, 4]. Type I tumors (30%) are low-grade, indolent tumors, while type II tumors (70%) are aggressive high-grade cancers that almost always present as advanced stage with high fatality [3, 5–8].

Dysbiosis is a pathological accommodation of the microbiome in compartments of an organism. Dysbiosis accompanies numerous diseases, among them neoplasias [9]. Dysbiosis accompanies ovarian cancer affecting, among others, the gut microbiome [10]. The healthy microbiome counteracts neoplastic cells through multi-pronged pathways in which the production of cytostatic metabolites play a prominent role [10] that was evidenced in ovarian cancer [11]. Cytostatic bacterial metabolites include compounds with various chemical structures, including bile acids.

Primary bile acids are synthetized in the liver [12]. Primary bile acids are secreted to the gastrointestinal tract to facilitate the emulsification of dietary fats and, hence, promote their digestion and uptake [12]. A fraction of primary bile acids are converted by intestinal bacteria to secondary bile acids through dihydroxylation and deconjugation [13]. Secondary bile acids are then absorbed from the gastrointestinal tract to the portal circulation to be subsequently cleared by the liver. Nevertheless, a fraction of the (secondary) bile acids remain in the systemic circulation and can elicit systemic effects similar to hormones [14]. Importantly, the ovary has a system for the production of bile acids [15, 16]. Mean follicular total bile acid level is around 10 µM [16], twice as the normal serum reference concentration.

Ursodeoxycholic acid (UDCA) is a secondary bile acid, its reference concentration in the human serum (conjugated+deconjugated UDCA) is between 100 and 300 nM [17–21]. In the study, we used UDCA in 300 nM that is the upper limit of the reference range [17–21]. The consideration behind 300 nM UDCA concentration is that in our previous studies biological effects were observed closer to the upper limit if the serum reference concentrations of bile acids [21–23] including UDCA [24], and, as noted above follicular bile acid levels are higher than the serum reference concentration ([16] vs [17–21]).

Bile acids can be found, besides the systemic circulation, in the ascites of ovarian cancer patients [25]. A deregulation of bile acid biosynthesis was observed in ovarian cancer patients [26–29]. Furthermore, multiple bile acid receptors were shown to be involved in the regulation of hallmarks of ovarian cancer (reviewed in [12]) pointing towards a possible involvement of bile acids in regulating the progression of ovarian cancer.

Among the secondary bile acids UDCA has a unique feature; while other secondary bile acids as lithocholic acid or deoxycholic acid can act as pro and anticarcinogenic agent as a function of the cancer, UDCA was shown by technically all studies to act as an anticarcinogenic agent (reviewed in [12]). Furthermore, UDCA can be used a formulated medication [30], therefore, UDCA can be (re)purposed for chemotherapy in ovarian cancer, a neoplasia with limited therapeutic options and short overall survival time [11]. Therefore, in this study, we assessed the effects of UDCA in cell models of ovarian cancer.

## Results

### Ursodeoxycholic acid induces EMT and cellular diapedesis

**Fig. 1:**
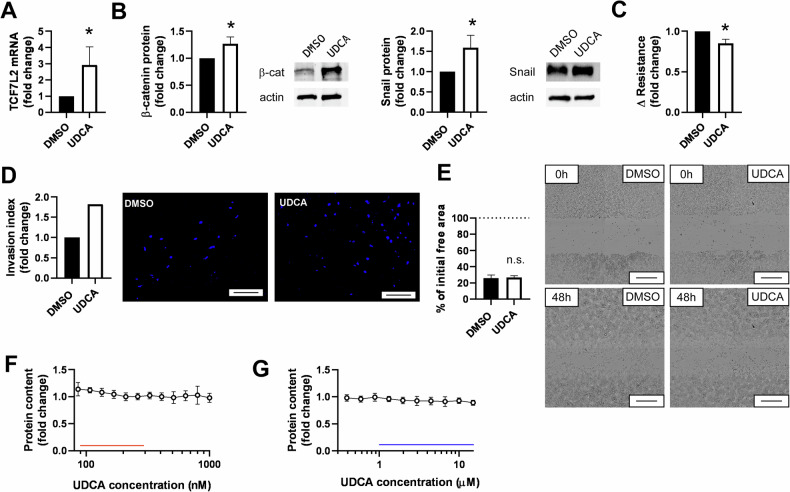
Ursodeoxycholic acid induces EMT in A2780 cells. **A** A2780 cells (8 × 10^4^/well) were plated to 6 well plates and were treated with 300 nM UDCA or DMSO for 48 h. Cells were scraped, RNA was prepared and the expression of the indicated gene was assessed by RT-qPCR. Three biological replicates are reported. **B** A2780 cells (5 × 10^5^) were plated into 10 cm Petri dish and were treated with 300 nM UDCA/DMSO for 48 h. Cells were scraped, total protein was prepared and lysates were subjected to SDS-PAGE and western blot. Blots were developed with the antibodies indicated and were evaluated by densitometry. Three biological replicates are reported. **C** A2780 cells (2 × 10^4^ or 5 × 10^4^) were plated into 8W10E ECIS plates (3–4 well/condition). After reaching confluency, cells were treated with 300 nM UDCA or DMSO as vehicle control. Changes to resistance was measured and calculated as described in the “Materials and methods”. Three biological replicates are reported. **D** A2780 cells (4 × 10^4^/well) were plated into the upper chamber of a Boyden chamber and were exposed to the chemoattractant for 24 h. The chamber was dismantled and the cells on the lower surface of the membrane were DAPI stained and counted. The invasion index was calculated and was expressed as fold change compared to the control. One biological replicate is reported. The scale bar equals 200 µm. **E** A2780 cells (5 × 10^4^ cells/well) were plated in 96-well plates and confluent cultures were generated. Scarring was performed using TECAN Freedom Evo robot. After scarring UDCA (300 nM)/DMSO (vehicle) treatment was initiated. Cell migration to the void area was monitored using the PE Opera Phoenix instrument for 48 h. Images were analyzed using the Image J software. The % of the original (T0) void area was expressed. The scale bar equals 500 µm. The assay was performed in quadruplicate; one biological replicate is presented. The dashed line represents the initial void area. **F**, **G** A2780 cells (10^3^ cells/well) were plated to 96 well plates and were treated with UDCA in the indicated concentrations for 48 h. On **F** concentrations correspond to the serum reference concentrations of UDCA (red line), on **G** concentrations correspond to the serum therapeutic concentrations of UDCA (blue line). Cell numbers were determined using the SRB assay. Treatments were performed in duplicates. On **F** 6, on **G** 8 biological replicates are reported. Data are represented as average ± SD with the exception of **D**, where no SD is presented. On **F**, **G** values were normalized for vehicle-treated cells, absorbance for vehicle-treated cells equals to 1. Normality was assessed using the Shapiro–Wilk test. Values were compared using unpaired *t*-test, except for (**F**, **G**). On **F**, **G** one-way ANOVA was performed, in the post-hoc test all values were compared to the vehicle control. * represent statistically significant difference between vehicle and UDCA-treated groups at *p* < 0.05. On **D**, **E** brightness and contrast were adjusted. CTL control, DMSO dimethyl-sulfoxide, n.s. not significant; TCF7L2 transcription factor 7-like 2, UDCA ursodeoxycholic acid.

Bile acids can influence epithelial-to-mesenchymal transition (EMT) in cell models of other cancers [21, 22, 31]. Therefore, we assessed whether UDCA has similar properties in the cell model of ovarian cancer. UDCA induced the expression of pro-EMT factors as the mRNA of Transcription factor 7-like 2 (TCF7L2) [32] and protein expression of β-catenin [33] and Snail [34] (Fig. 1A, B) suggesting that UDCA induces EMT. In line with these expressional changes, UDCA reduced the adherence of cells to each other and to the surface as shown in impedance measurements (Fig. 1C). Furthermore, UDCA supported chemotaxis and diapedesis of ovarian cancer cells in the Boyden chamber (Fig. 1D). However, UDCA did not influence the behavior of cells in an in vitro model of wound healing (Fig. 1E) and did not impact on the rate of cell proliferation when applied either in the reference concentration range (Fig. 1F) or in concentrations in the therapeutic range (Fig. 1G).

**Fig. 2:**
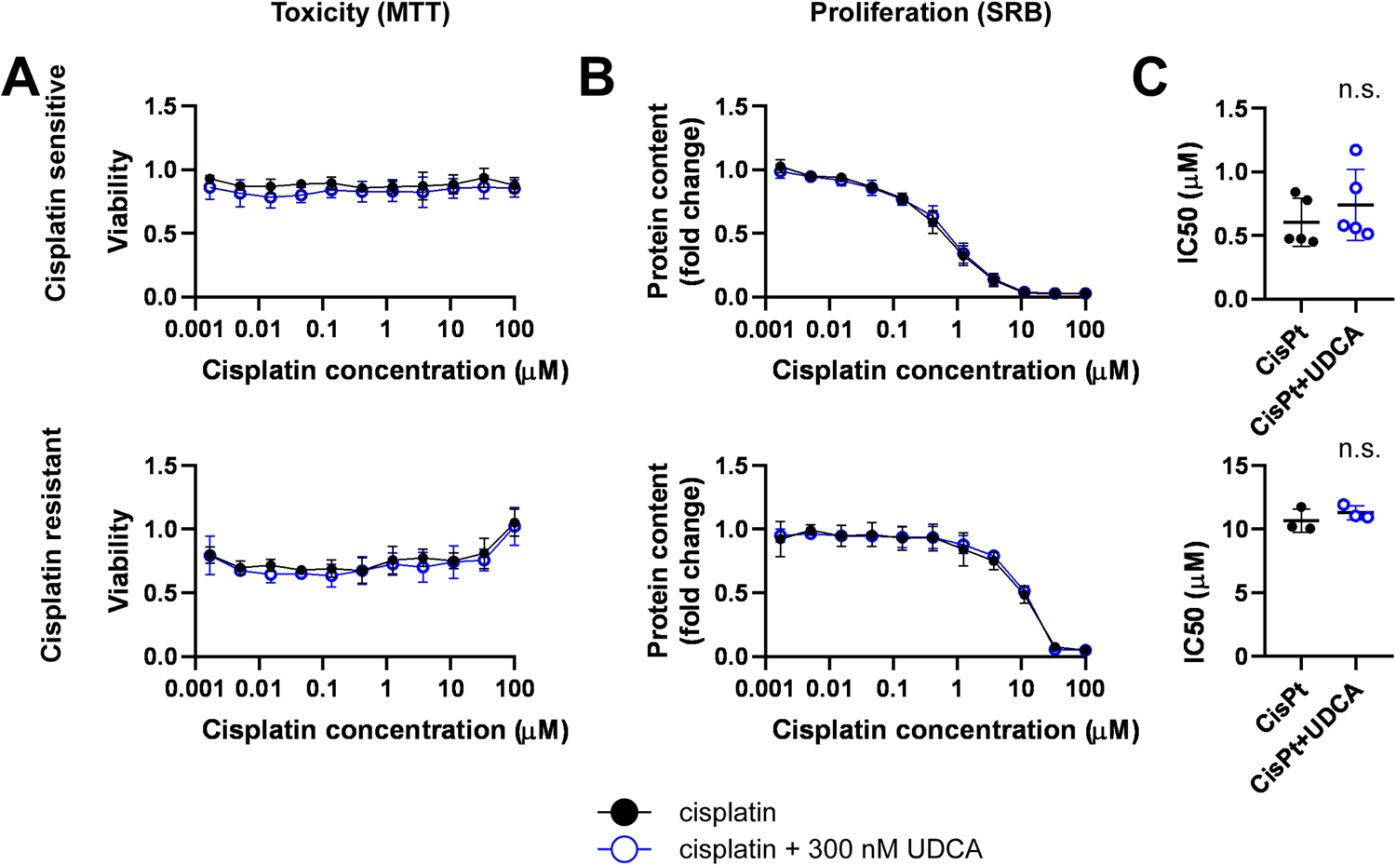
Ursodeoxycholic acid does not influence the cisplatin sensitivity of A2780 cells. **A**–**C** A2780 cells were plated in 96 well plates (10^4^ cells/well for the MTT assay for both cell lines; for SRB 2 × 10^3^ cisplatin sensitive cells/well and 6 × 10^3^ cisplatin resistant cells/well) and were treated with cisplatin as indicated with or without 300 nM UDCA. In the MTT assay (**A**) the treatment lasted 4 h, in the SRB assay (**B**) the treatment lasted 48 h. The IC50 values obtained from the SRB assay are depicted on (**C**). Data are presented as means ± SD. Except for the SRB assay on cisplatin-sensitive cells, where 5 biological replicates are presented, all other assays were performed in 3 biological replicates. Individual assays were measured in duplicates. Values were normalized to vehicle-treated cells (absorbance is equal to 1). Nonlinear regression (GraphPad “[Inhibitor] vs. response (four parameters)” utility) was performed on datasets to obtain IC50 values. Normality of the IC50 values were determined using the Shapiro-Wilk test. Statistical difference between the inhibitory curves was performed non-paired, two-sided t-test was applied. CisPt cisplatin, MTT 3-(4,5-dimethylthiazol-2-yl)-2,5-diphenyltetrazolium bromide, SRB sulforhodamine B, UDCA ursodeoxycholic acid.

EMT can support drug resistance of cancer cells, among them, ovarian cancer cells [35]. As the therapy of ovarian cancer is built on platinum-based drugs [11], we assessed the sensitivity of A2780 cells and A2780 cisplatin-resistant cells to cisplatin with and without UDCA treatment in a short timeframe (4 h, MTT assay; Fig. 2A) and in a long timeframe (48 h, SRB assay; Fig. 2B, C) (similar to [36]). Cisplatin had no effect in the short timeframe neither in cisplatin-sensitive nor in cisplatin-resistant cells (Fig. 2A), in contrast to the long treatment scheme (Fig. 2B, C). However, UDCA treatment did not affect cisplatin-induced cytostasis (Fig. 2B, C).

### Ursodeoxycholic acid induces NRF2 expression and reduces oxidative stress

Bile acids in other cancer models impacted on cancer cell proliferation through mediating the redox status of cancer cells (e.g. [21, 23, 37, 38]). Changes to the redox status of cells upon bile acid treatment depends on changes to the expression and cellular localization of nuclear factor erythroid 2-related factor 2 (NRF2/NFE2L2) [39, 40].

**Fig. 3:**
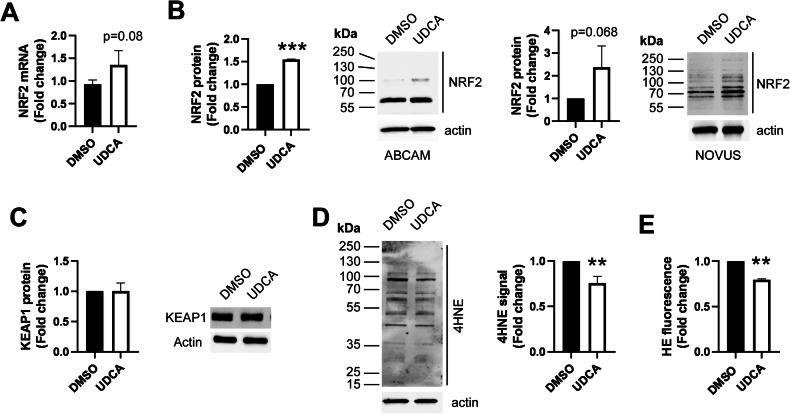
Ursodeoxycholic acid induces NRF2 expression and reduces oxidative stress, but does not modulate cell proliferation. **A** A2780 cells (10^5^/well) were plated in 6-well plates and were treated with UDCA or DMSO as vehicle for 48 h. Cells were scraped, RNA was prepared and the expression of Nrf2 gene was assessed by RT-qPCR. Three biological replicates are reported. **B**–**D** A2780 cells were plated in 6 cm petri dishes (10^6^ cells/dish) and were treated with 300 nM UDCA or DMSO as vehicle for 48 h. Total protein was prepared and lysates were subjected to SDS-PAGE and Western blot. Blots were developed with the antibodies indicated and were evaluated by densitometry. Number of biological replicates on **B** is 2 for the Novus and 2 for the Abcam antibody; on **C**, the number of biological replicates is 4; on **D**, the number of biological replicates is 3. **E** A2780 cells were plated in 24 well plates (10^4^ cells/well) and were treated with 300 nM UDCA or DMSO as vehicle control for 48 h. Cells were stained with 2.5 µM Hydroethidine for 30 min and fluorescence signal was measured by flow cytometry. Treatments were performed in 6 replicates. Result from 2 independent experiments is reported. All values are depicted as mean ± SD. The number of biological replicates is the following: **B** Abcam *n* = 2, Novus *n* = 2, **C** *n* = 4, **D** *n* = 3. Normality was assessed using the Shapiro–Wilk test. Densitometric values were compared using unpaired *t*-test. ** and *** indicate statistical difference between vehicle and UDCA-treated cells at *p* < 0.01 or *p* < 0.001, respectively. DMSO dimethyl sulfoxide, HE hydroethidine, KEAP1 Kelch-like ECH-associated protein 1, NRF2 nuclear factor erythroid 2-related factor 2, UDCA ursodeoxycholic acid, 4HNE 4-hydroxy-nonenal.

UDCA induced the mRNA (Fig. 3A) and protein expression of NRF2 (Fig. 3B) without influencing the expression of Kelch-like ECH-associated protein 1 (KEAP1) (Fig. 3C) that is an intrinsic inhibitor of NRF2. These suggest the induction of NRF2 and in line with that, we observed a reduced immunosignal of 4-hydroxy-nonenal (4HNE), an oxidative stress marker [41] (Fig. 3D) and reduced hydroethidine signal, an indication of lower superoxide production [42] (Fig. 3E). UDCA treatment yielded 4HNE signals throughout the whole molecular weight range of the lysate (Fig. 3D), in contrast to our previous observations, where rather well-defined protein targets were identified.

### Ursodeoxycholic acid induces glycolytic flux in A2780 cells

Cancer cells are characterized by changes to metabolism [43–45]. Bile acids [12, 14, 21] and, more specifically, UDCA [46–48] can induce oxidative metabolism raising the possibility of UDCA-elicited metabolic changes in A2780 cells. Similar, NRF2 activation and the subsequent change to cellular redox homeostasis can also induce changes to intermediary metabolism [39, 49–55].

**Fig. 4:**
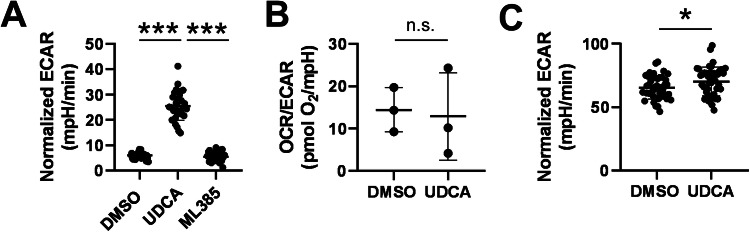
UDCA induces glycolysis through activating NRF2. **A**, **B** A2780 cells (6 × 10^3^ cells/well) were plated into Seahorse plates and were treated with the indicated chemicals or DMSO as vehicle for 48 h. The Seahorse assay was performed as described in the “Materials and methods”. On **A**, one typical experiment is shown, on **B**, three experiments are shown. **C** ID8 cells (5 × 10^3^ cells/well) were plated into Seahorse plates and were treated with UDCA or DMSO as vehicle for 48 h. The Seahorse assay was performed as described in the “Materials and methods”. All values are depicted as mean ± SD. Each well was considered separate during the calculations. Normality was assessed using the Shapiro-Wilk test and on **A**, one-way ANOVA was performed followed by a post-hoc test comparing all combinations of cohorts, while on **B**, **C** unpaired *t*-test was performed. * and *** indicate statistically significant difference between the cohorts at *p* < 0.05 and 0.001, respectively. DMSO dimethyl sulfoxide, ECAR extracellular acidification rate, n.s. not significant, UDCA ursodeoxycholic acid.

UDCA induced the ECAR value in A2780 cells (Fig. 4A) suggesting increases in glycolytic flux. In turn, UDCA did not modulate oxidative phosphorylation (assessed through the OCR value) (Fig. 4B). The induction of glycolysis was dependent on the activation of NRF2, as a pharmacological inhibitor of NRF2, ML385 [56] inhibited the induction of ECAR (Fig. 4A).

Finally, we assessed whether the induction of ECAR by UDCA can be generalized to other ovarian cancer cell lines. The treatment of ID8 murine ovarian cancer cells with UDCA led to an induction of ECAR (Fig. 4C), similar to the observations on A2780 cells.

**Fig. 5:**
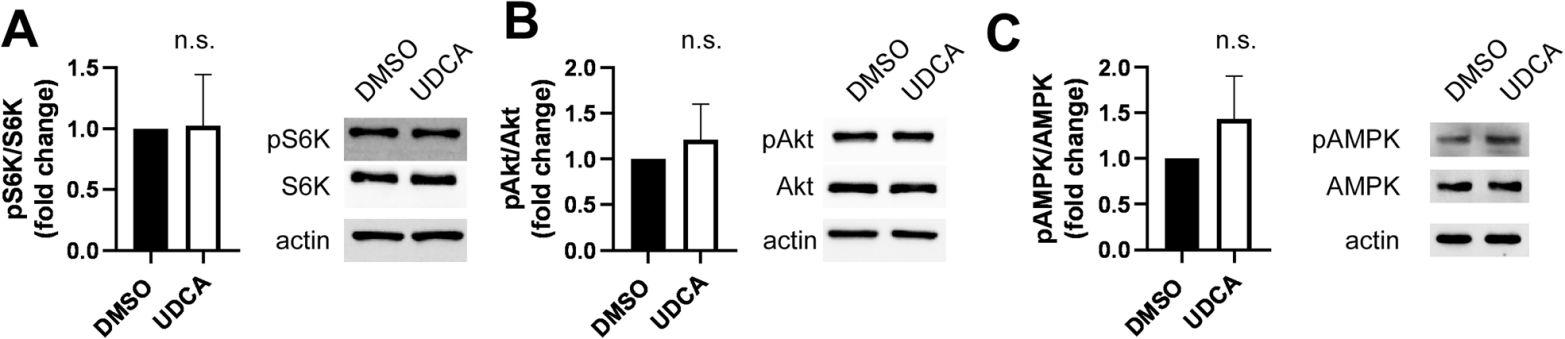
Ursodeoxycholic acid does not interfere with the AMPK/mTOR system. **A**–**C** A2780 cells were plated into 6 cm petri dishes (10^6^/dish) and were treated with 300 nM UDCA or DMSO as vehicle control for 48 h. Cells were scraped, total protein was prepared and lysates were subjected to SDS-PAGE and Western blot. Blots were developed with the antibodies indicated and were evaluated by densitometry. All values are depicted as mean ± SD. Normality was assessed using the Shapiro-Wilk test. Densitometric values were compared using unpaired *t*-test. The following number of biological replicates were assessed: pS6K/S6K: 6, pAkt/Akt: 5, pAMPK/AMPK: 4. AMPK AMP-activated kinase, DMSO dimethyl sulfoxide, UDCA ursodeoxycholic acid, p70 S6K p70 S6 kinase.

In addition, we assessed the activity of the AMP-activated kinase (AMPK) and the mechanistic target of rapamycin (mTOR) system in response to UDCA. The phosphorylation of p70 S6 kinase (S6K) at Thr389 was used as a proxy to assess the activity of mTOR complex 1 (mTORC1) and the phosphorylation of Akt kinase at Ser473 was used as a proxy to assess the activity of mTOR complex 2 (mTORC2). AMPK activity was judged by assessing an activating autophosphorylation site of AMPK (at Thr172). UDCA treatment did not influence the level of phosphorylation of S6K (Fig. 5A), Akt (Fig. 5B) and AMPK (Fig. 5C) suggesting that the activity of the AMPK/mTOR system is not influenced by UDCA treatment.

### The induction of ursodeoxycholic acid-dependent genes associate with worse clinical outcome

**Fig. 6:**
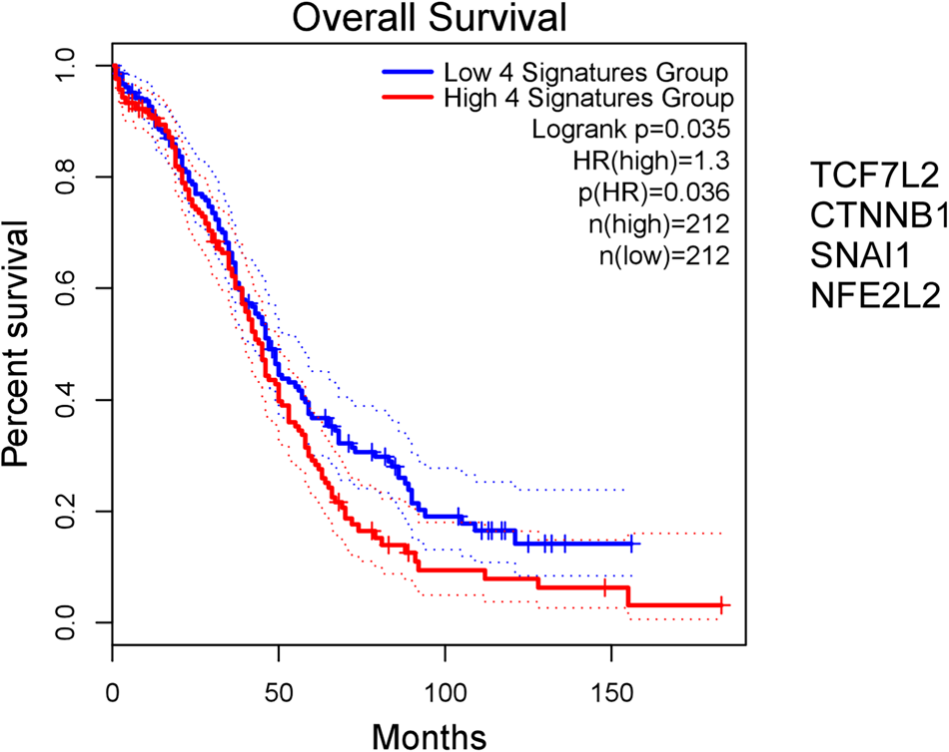
The tumoral overexpression of the subset of the ursodeoxycholic acid-induced genes correlate with worse clinical outcomes in humans. The GEPIA2 database was assessed for correlation between patient survival and composite expression of the indicated gene set. All databases were accessed on the 23 August 2024. CTNNB1 β-catenin, HR hazard ratio, NFE2L2 nuclear factor erythroid 2-related factor 2, TCF7L2 transcription factor 7-like 2.

In the study we identified a set of genes that were induced by UDCA either that level of mRNA/protein expression or at the level of activity (TCF7L2, CTNNB1, SNAI1 and NFE2L2). We assessed the composite effect of the 4 gene signature on patient survival using the GEPIA2 database. The tumoral overexpression of the 4 gene signature correlated with markedly worse survival with a hazard ratio of 1.3 (Fig. 6).

## Discussion

In this study we showed that UDCA, when administered in concentrations corresponding to the human serum reference concentration, promotes cancer progression. In previous studies on a plethora of different neoplasias UDCA exerted beneficial, antineoplastic effects (reviewed in [12]) by inducing apoptosis or supporting chemosensitization [57–59]. In this regard, our observations are novel and surprising.

UDCA exerted multi-pronged effects by impacting on EMT, cell adhesion, diapedesis, glycolytic dominance and oxidative stress. We observed the complex effects of bile acids in cell models of other neoplasia [21, 31] similar to previous studies on ovarian cancer [57–59]. The only contradiction among this study and previous observations is the lack of cytotoxicity or cytostasis in our data contrasted to prior art [58, 59], however, this is likely a concentration-dependent difference. Bile acids in high concentration can prove toxic, as bile acids can harm biomembranes, however, in submicromolar concentrations this is not likely (e.g. [21]).

Serum primary and secondary bile acid levels, including glycoursodeoxycholic acid, decrease in ovarian cancer patients [26, 27]. Furthermore, bile acid biosynthesis [60–62], similar to other metabolites [63–65], decreases with age and age is a risk factor for ovarian cancer. These observations make internal ursodeoxycholic overproduction in patients unlikely, however, UDCA supplementation as medication may increase the risk of progression in patients with ovarian cancer.

We identified NRF2 overexpression as a mechanism of UDCA-induced changes. NRF2 overexpression [66, 67] supports cancer progression [39, 44], metastasis formation, induce EMT [68, 69] and glycolytic flux [39, 49–55]. Reduction of 4HNE signal upon UDCA treatment points out a reduction in naturally occurring oxidative stress that can be considered reductive stress [70]. Of note, reductive stress rearranges cellular intermediary metabolism and supports the Warburg-type metabolism [55] that we observed in UDCA-treated cells hereby. Warburg rearrangement occurs in ovarian cancer cells [71–75], and changes to cellular metabolism are associated with metastatic capacity [74–83] suggesting similar properties for UDCA.

We assessed the possible involvement of the AMPK/mTOR system. The dysregulation of the AMPK/mTOR system is frequent in neoplasias [84, 85] and the activation of mTOR frequently leads to the induction of glycolysis in cancers including ovarian cancer [86, 87]. In light of the importance of glycolytic induction and mTOR activation in supporting cell proliferation in ovarian cancer [86–88], the absence of changes to the activity of the AMPK/mTOR system is surprising.

Also to our surprise, we have not observed the induction of chemoresistance upon UDCA treatment neither in cisplatin resistant, nor in cisplatin sensitive A2780 cells, despite the widespread literature reports on resistance against numerous chemotherapy agents upon NRF2 induction in multiple cancers, including ovarian cancer [57, 59, 89–92].

Hereby, we provided evidence that UDCA treatment in cellular models of ovarian cancer induces features of cancer progression and metastasis formation. High tumoral expression of the UDCA-induced genes correlate in humans with shorter survival suggesting that increases in UDCA concentration in the environment of ovarian cancer cells support cancer progression. These observations are in conflict with the prior observations on the widespread antineoplastic effects UDCA in multiple cancers [12], on a broader scale that points out that bacterial metabolites have cancer-specific actions and may have opposing features in different neoplasias. Furthermore, our results point towards a cautious administration of UDCA-containing drugs in ovarian cancer patients.

## Materials and methods

### Chemicals

All chemicals were purchased from Sigma-Aldrich (St. Louis, MO, USA) unless stated otherwise. The sources of key chemicals are assembled in Table 1. UDCA was purchased from Sigma-Aldrich (St. Louis, MI, USA) and was dissolved in dimethyl sulfoxide (DMSO) to achieve the final concentration of 100 mM. Cisplatin was dissolved in 1xPBS at the concentration of 3 mM, ML385 was dissolved in DMSO in the concentration of 50 mM.

**Table 1:** The source of key chemicals.

Compound	Company	Ref. No.
UDCA	Sigma-Aldrich 3050 Spruce Street, Saint Louis, MO 63103, USA	U5127 CAS 128-13-2
Cisplatin	Sigma-Aldrich 3050 Spruce Street, Saint Louis, MO 63103, USA	232120 CAS 15663-27-1
ML385	Selleck Chemicals 14408W Sylvanfield Drive, Houston, TX 77014 USA	S8790 CAS 846557-71-9

### Cell lines

*A2780* cells were cultured in RPMI 1640 medium supplemented with 10% fetal calf serum, 2 mM l-glutamine, 1% penicillin-streptomycin.

*ID8* cells were cultured in high glucose DMEM (4.5 g/L glucose) medium supplemented with 4% fetal calf serum, 2 mM L-glutamine, 1% penicillin–streptomycin, 1% ITS supplement (I3146).

*Cisplatin-resistant A2780* cells were grown in RPMI 1640 medium supplemented with 10% fetal calf serum, 2 mM glutamine, 1% penicillin-streptomycin. Cisplatin-resistant cells underwent selection (1 µM cisplatin) once a week for 3 days before plating for assay.

Cell lines were regularly checked for mycoplasma contamination.

### Methylthiazolyldiphenyl-tetrazolium bromide (MTT) reduction assay

MTT reduction assay was measured as previously described [36]. The assay determines the activity of mitochondrial complex I and can be used to detect rapid toxicity and the induction of apoptosis [93]. Briefly, cells were plated in 96 well plates the day before the assay. A2780 cells or cisplatin-resistant A2780 cells were plated in 96 well plates for MTT assays with a number of 10^4^ cells/well. On the next day, cells were treated with cisplatin in itself or in a combination with 300 nM UDCA for 4 h. Cisplatin was applied between the concentrations of 100 µM and 0.0017 µM with one-third dilution. Vehicle controls were DMSO for UDCA and 1xPBS for cisplatin.

### Sulforhodamine B assay

The SRB assay measures acid-precipitable protein onto cells that is proportional to cell number, hence can be used to assess cell proliferation or long-term cytostasis [94]. SRB proliferation assay was measured as previously described [36].

For Fig. 1, A2780 cells (10^3^ cells/well) were plated to 96-well plates and were treated with UDCA on the next day for 48 h. For concentration range of serum reference, UDCA was applied between 1000 nM and 86 nM with four-fifths dilution. For therapeutic concentration range UDCA was applied between 15 µM and 0.4 µM with two-thirds dilution.

For Fig. 2, A2780 cells (2 × 10^3^ cells/well) and cisplatin-resistant A2780 cells (6 × 10^3^ cells/well) were plated in 96 well plates. On the next day, cells were treated with cisplatin in itself or in a combination with 300 nM UDCA for 48 h. Cisplatin was applied between the concentrations of 100 µM and 0.0017 µM with one-third dilution. Vehicle controls were DMSO for UDCA and 1xPBS for cisplatin.

### Impedance measurements

Electric cell-substrate impedance sensing (ECIS Z-Theta, Applied BioPhysics) was applied as described previously [95]. A2780 cells (2 × 10^4^ or 5 × 10^4^) were plated into 8W10E ECIS plates (3-4 well/condition). This plate is recommended to monitor cell-cell connections. Impedance measurement was initiated upon plating and was continued onwards. Impedance was measured at multiple frequencies between 62.5 Hz and 64 kHz in 120 or 150 s (as a function of cell numbers) time intervals in real time. After reaching confluency (plateau in capacitance and resistance), half of the medium was replaced by 600 nM UDCA (yielding a final concentration of 300 nM) or DMSO as vehicle control. The impedance value corresponding to the plateau (i.e. confluence) was regarded as a baseline value. Impedance measurement was resumed for 24 h. The average of the resistance values at 4 kHz ±30 min before and after the peak resistance was regarded as a peak value. The difference between the peak and the baseline values were expressed as fold change compared to the DMSO-treated samples and were plotted. All plates contained a well containing only medium as a background control for background correction.

### Western blot

Cells were lysed in RIPA buffer (50 mM Tris, 150 mM NaCl, 0.1% SDS, 1% TritonX 100, 0.5% sodium deoxycolate, 1 mM EDTA, 1 mM Na_3_VO_4_, 1 mM NaF, 1 mM PMSF, protease inhibitor coctail). Protein concentrations were determined using a BCA protein assay kit (Pierce Biotechnologies, Rockford, IL, USA). Protein extracts were loaded onto 10% SDS polyacrylamide gels. After electrophoresis, proteins were transferred to nitrocellulose membranes and blocked using 5% BSA for 1 h at room temperature. Then, membranes were incubated with primary antibodies for overnight at 4 °C. Membranes were washed with Tris-buffered saline containing 0.1% Tween (TBS-T) followed by a 1 h incubation with IgG HRP conjugated secondary antibody (Cell Signaling Technology, Inc. Beverly, MA, 1:2000). Membranes were washed with TBS-T and visualization was performed by SuperSignal West Pico Solutions (Thermo Fisher Scientific). β-actin was used as a loading control. Blots were quantified by densitometry using Image Lab 6.1 software.

Antibodies used in this study are listed in Table 2.

**Table 2:** The antibodies used in the study.

Name	Species	Ig class	Dilution	Vendor and Cat No.	RRID
Actin	Rabbit pAb		1:30000	Sigma-Aldrich A2066	AB 476693
Akt	Rabbit pAb		1:1000	Cell Signaling Technology #9272	AB 329827
Phospho-Akt (Ser473) (D9E) XP®	Rabbit mAb	IgG	1:2000	Cell Signaling Technology #4060	AB 2315049
AMPKα (D63G4)	Rabbit mAb		1:1000	Cell Signaling Technology #5832	AB 10624867
Phospho-AMPKα (Thr172) (40H9)	Rabbit mAb	IgG	1:1000	Cell Signaling Technology #2535	AB 331250
KEAP1 (D6B12)	Rabbit mAb	IgG	1:1000	Cell Signaling Technology #8047	AB 10860776
Nrf2	Rabbit pAb	IgG	1:1000	Abcam ab31163	AB 881705
Nrf2	Rabbit pAb	IgG	1:1000	Novus Biologicals NBP1-32822	AB 10003994
p70 S6 Kinase (49D7)	Rabbit mAb	IgG	1:1000	Cell Signaling Technology #2708	AB_390722
Phospho-p70 S6 Kinase (Thr389)	Rabbit pAb		1:1000	Cell Signaling Technology #9205	AB 330944
Snail (C15D3)	Rabbit mAb	IgG	1:1000	Cell Signaling Technology #3879	AB 2255011
4 Hydroxynonenal	Rabbit pAb	IgG	1:1000	Abcam ab46545	AB_722490
β-Catenin (D10A8) XP®	Rabbit mAb	IgG	1:1000	Cell Signaling Technology #8480	AB_11127855
Anti-rabbit IgG HRP-linked	Goat	IgG	1:10000	Cell Signaling Technology #7074	AB_2099233

*mAb* monoclonal antibody, *pAb* polyclonal antibody.

### Total RNA preparation and reverse transcription-coupled quantitative PCR (RT-qPCR)

Total RNA from A2780 cells was prepared using TRIzol reagent (cat. no. TR118, Molecular Research Center Inc., Cincinnati, OH, USA) according to the manufacturer’s instructions. DNase treatment of total RNA samples was performed using 2U of DNaseI (cat. no. AM2222, Thermo Fisher Scientific) per 10 µg RNA. Two micrograms of total RNA were used for reverse transcription using High-Capacity cDNA Reverse Transcription Kit (cat. no. 4368813, Thermo Fisher Scientific) according to the manufacturer’s instructions. The reverse transcription reaction mixtures were supplemented with RNase inhibitor (cat. no. N8080119, Thermo Fisher Scientific). The cDNA was cleaned with the nucleospin Gel and PCR clean-up (Macherey-Nagel Gmbh & Co. KG, Düren, Germany) according to the manufacturer’s instructions.

The qPCR reactions were performed with qPCRBIO SyGreen Lo-ROX Supermix (PCR Biosystems Ltd., London, UK) using 20 ng diluted cDNA with the appropriate primers at 500 nM final concentration in 10 μl total volume. Amplification reactions were performed using a Light-Cycler 480 system (Roche Applied Science, Basel, Switzerland). Cycling conditions are given in Table 3. Primers are listed in Table 4.

**Table 3:** Cycling conditions of qPCR reactions.

Step	Temperature	Time	Cycles
Polymerase activation	95 °C	10 min	1
Denaturation	95 °C	10 s	50
Annealing/Extension	62 °C	20 s	
Melting curve analysis	95 °C	5 s	1
	65 °C	1 min	
	65 °C to 97 °C (Continuous)	Ramp rate 0.11 °C/s	
	40 °C	30 s

**Table 4:** The specifics of the primer sets used in the study.

Gene name	Primers (5′-3′)	Length (bp)	Specificity check	Alignments (Sequence ID) Query coverage 100% Percent identity 100%	Primer location	Exon intron boundary	Calibration curve	Efficiency
NRF2	FW: TGAGCAAGTTTGGGAGGAGCT REV: ACTGGTTGGGGTCTTCTGTGG	245	BLAST	NM_001313903.2	FW 484-504 REV 728-708	YES	Slope: −3.428 YIntercept: 28.11 Error = 0.0735	1.958
				NM_001313902.2	FW 613-633 REV 857-837			
				NM_006164.5	FW 703-723 REV 947-927			
				NM_001313904.1	FW 1165-1185 REV 1409-1389			
				NM_001313901.1	FW 1202-1222 REV 1446-1426			
				NM_001313900.1	FW 1110-1130 REV 1354-1334			
				NM_001145413.3	FW 1215-1235 REV 1459-1439			
				NM_001145412.3	FW 1236-1256 REV 1480-1460			
TCF7L2	FW: ACGTACAGCAATGAACACTTCAC REV: GGCGATAGTGGGTAATACGG	128	BLAST	NM_001198530.2	FW 952-974 REV 1079-1060	YES	Slope: -3.699 YIntercept: 29.42 Error = 0.204	1.863
				NM_001146283.2	FW 1195-1217 REV 1322-1303			
				NM_001349870.2	FW 778-800 REV 905-886			
				NM_001146285.2 NM_001198528.2 NM_001146286.2 NM_001198527.2 NM_001198526.2 NM_001146284.2 NM_001198525.2 NM_001198529.2 NM_030756.5	FW 1054-1076 REV 1181-1162			
				NM_001367943.1 NM_001146274.2 NM_001363501.2 NM_001198531.2	FW 1123-1145 REV 1250-1231			
				NM_001349871.1	FW 310-332 REV 437-418			
PPIA	FW: GTCTCCTTTGAGCTGTTTGCAGAC REV: CTTGCCACCAGTGCCATTATG	171	BLAST	NM_021130.5	FW 102-125 REV 272-252	YES	Slope: -3,485 YIntercept: 25.54 Error= 0.0366	1.936
RPLP0	FW: CCATTGAAATCCTGAGTGATGTG REV: GTCGAACACCTGCTGGATGAC	131	BLAST	NM_053275.4	FW 589-611 REV 719-699	YES	Slope: -3.552 YIntercept: 30.65 Error= 0.0548	1.912
				NM_001002.4	FW 529-551 REV 659-639			

Reporting is made in accordance with the QIME guideline [97, 98].

### Boyden chamber experiments

Cell invasion assay was determined using Corning BioCoat Matrigel Invasion Chambers (Corning, NY, USA) with 8.0 µm PET membrane in 24-well plates (cat.no.# 354480). In the control setup similar 8 µm PET membranes were used that were devoid of the matrigel layers. Cells (20.000 cells/well) were added to the top of insert in upper chamber in serum free medium and were seeded overnight. Then, cells were treated with UDCA (300 nM) in 0.5 ml serum-free medium. Simultaneously medium (0.75 ml) containing 10% FBS, UDCA (300 nM) and 100 ng/ml stromal cell-derived factor 1α (SDF1α) (Sigma-Aldrich, cat.no.# SRP4388) was added to the lower chamber as chemoattractant. After 48 h, non-invaded cells on the top of the filters were wiped off with cotton swabs. The lower surface of membranes containing invaded cells were washed in PBS and fixed with 100% methanol and stained with DAPI. The invading cells were counted with Opera Phoenix High Content Screening System (Perkin-Elmer Waltham, MA, USA) and pictures were analyzed using the Harmony 4.6 Software. The following calculations were performed:

% Invasion = (Mean of cells invading through Matrigel insert membrane/Mean of cells invading through Control insert membrane) * 100

Invasion index = % Invasion of the Treated cell / % Invasion of the Control (non-treated) cell

### Seahorse measurements

A2780 (6 × 10^3^) or ID8 (5 × 10^3^) cells were plated into Seahorse XF96 cell culture microplates (Agilent Technologies, Inc., Santa Clara, CAL, USA) and were treated with 300 nM UDCA or DMSO (as vehicle). In a subset of experiments UDCA-treated cells were treated with ML385 (1 µM) for 48 h. At the end of treatment medium was replaced by buffer-free DMEM supplemented with glucose (1 g/L) and the plate was incubated in a CO_2_-free incubator for at least 1 h and then the plate was subjected to Seahorse measurement using the Seahorse XF96 instrument. Five-minute measurements were made that was repeated 5 times, interspersed by 30 s of mixing. After the assay cell numbers were determined by SRB assay. The stable ECAR readouts were averaged for each well, the first measurement point was omitted. The ECAR values were normalized for the SRB absorbance.

### Database screening

We assessed the survival data in GEPIA2 [96] and database.

### Statistical analysis

Statistical analysis was performed using 8.0.1 version of Graphpad Prism. Values were tested for normal distribution using the test indicated in the figure legends. When necessary, values were log normalized. The following statistical test, post hoc test and the level of significance is indicated in the figure captions. Nonlinear regression was performed using the built-in “[Inhibitor] vs. response—Variable slope (four parameters), least square fit” utility of Graphpad that yielded IC50 and Hill slope values.

## Supplementary information

Supplementary Material

## Data Availability

Primary data are available at figshare.com (https://figshare.com/s/1f7e89f1c6e4cd82749d; 10.6084/m9.figshare.26825533).
