# Supplementary material for: Ursodeoxycholic acid prompts glycolytic dominance, reductive stress and epithelial-to-mesenchymal transition in ovarian cancer cells through NRF2 activation
Source: Cell Death Discov. 2025 Apr 3;11:134. doi: 10.1038/s41420-025-02398-9 (PMC11965337; doi:10.1038/s41420-025-02398-9)

Figure 1. panel B: β-catenin

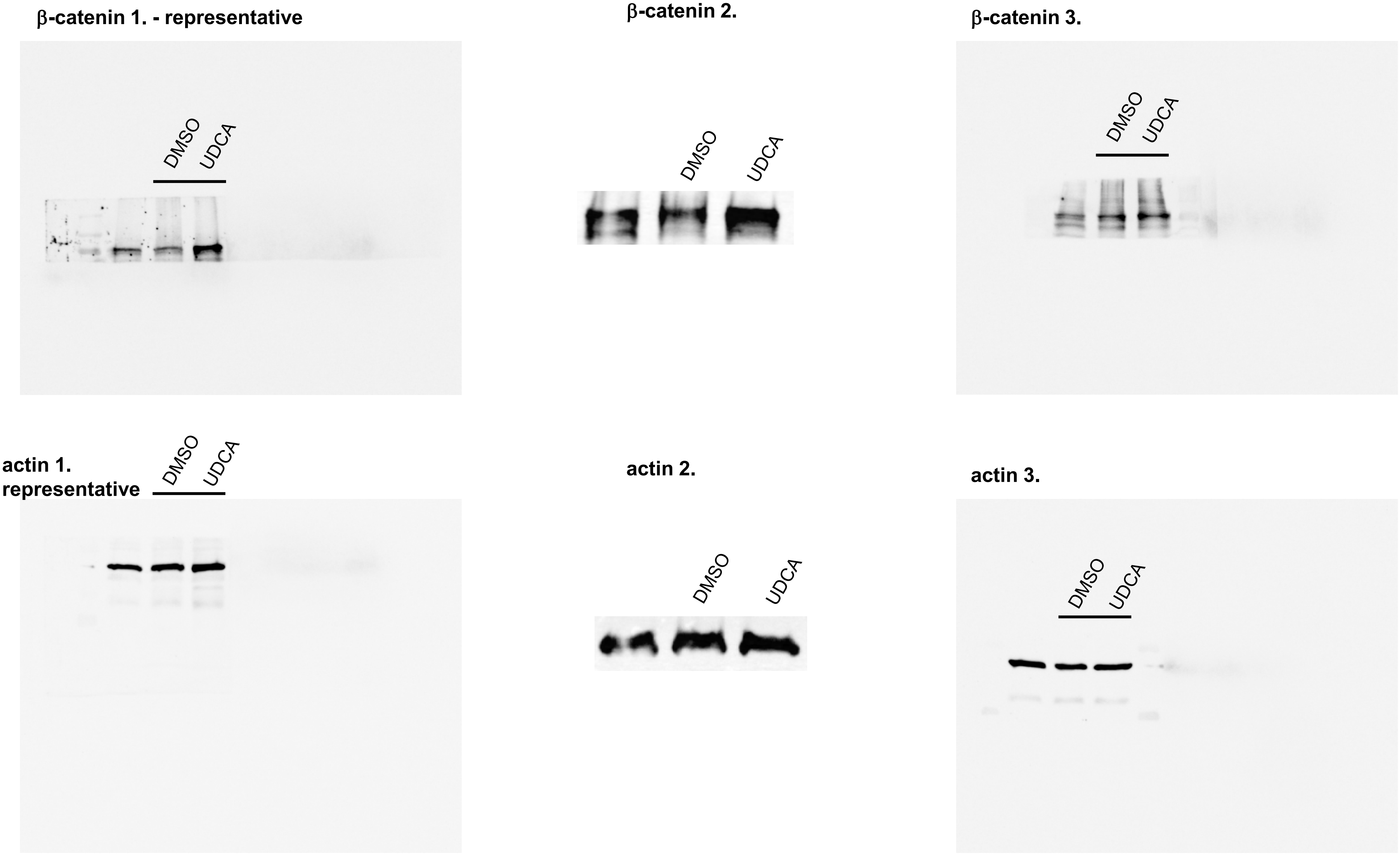

Figure 1. panel B: Snail

Snail 1.

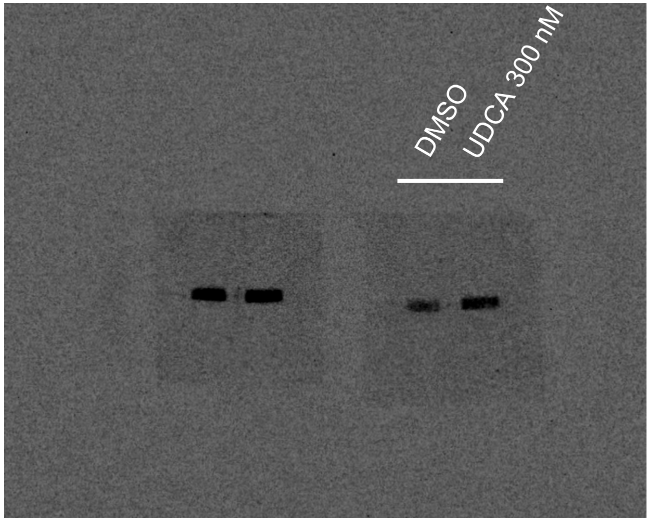

Snail 2.

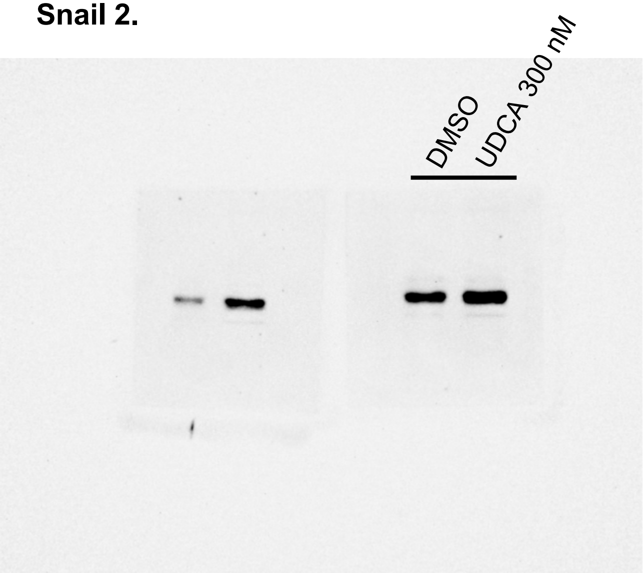

Snail 3. - representative

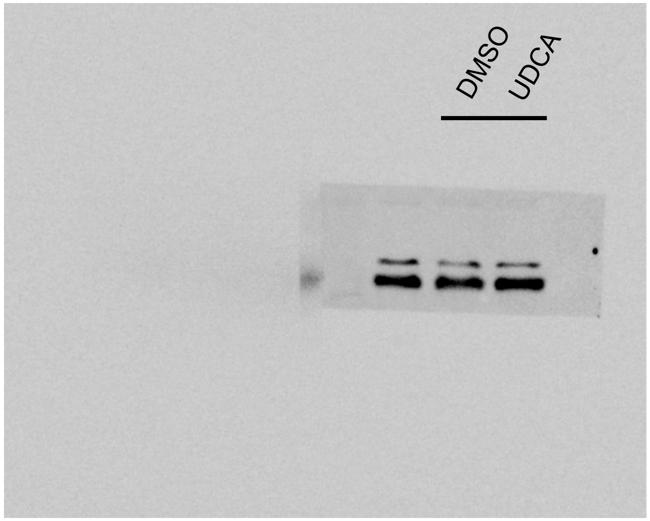

actin 1.

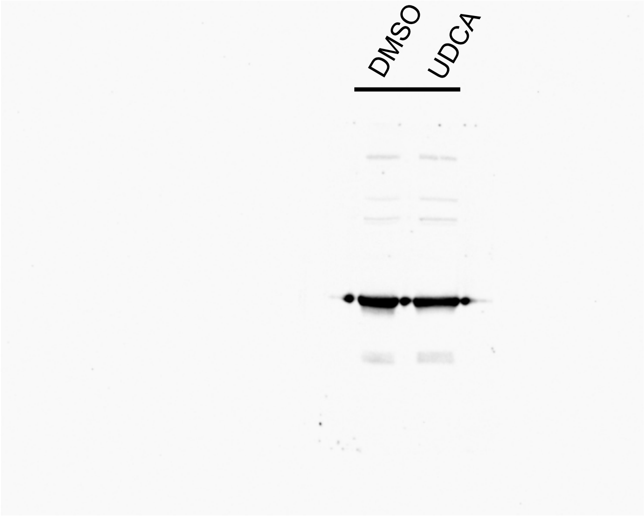

actin 2.

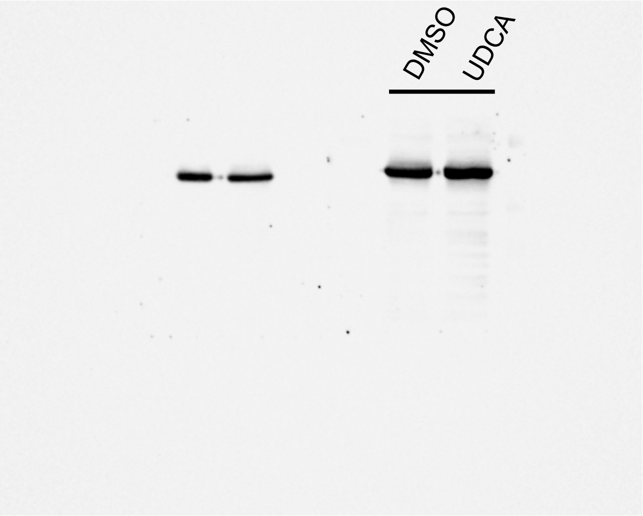

actin 3. – representative

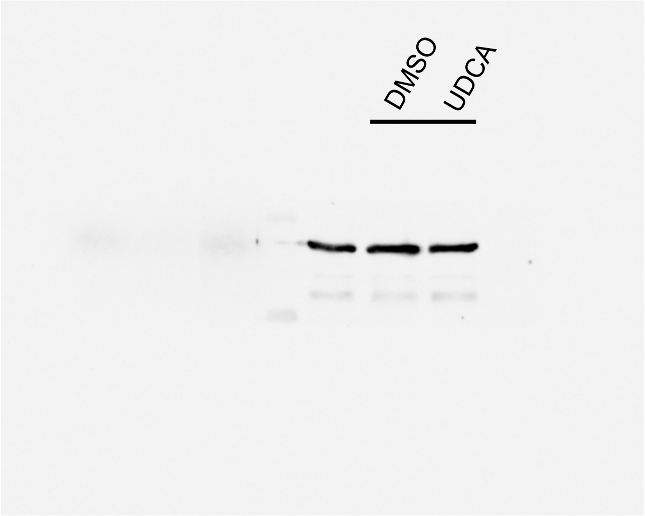

Figure 3. panel B: NRF2

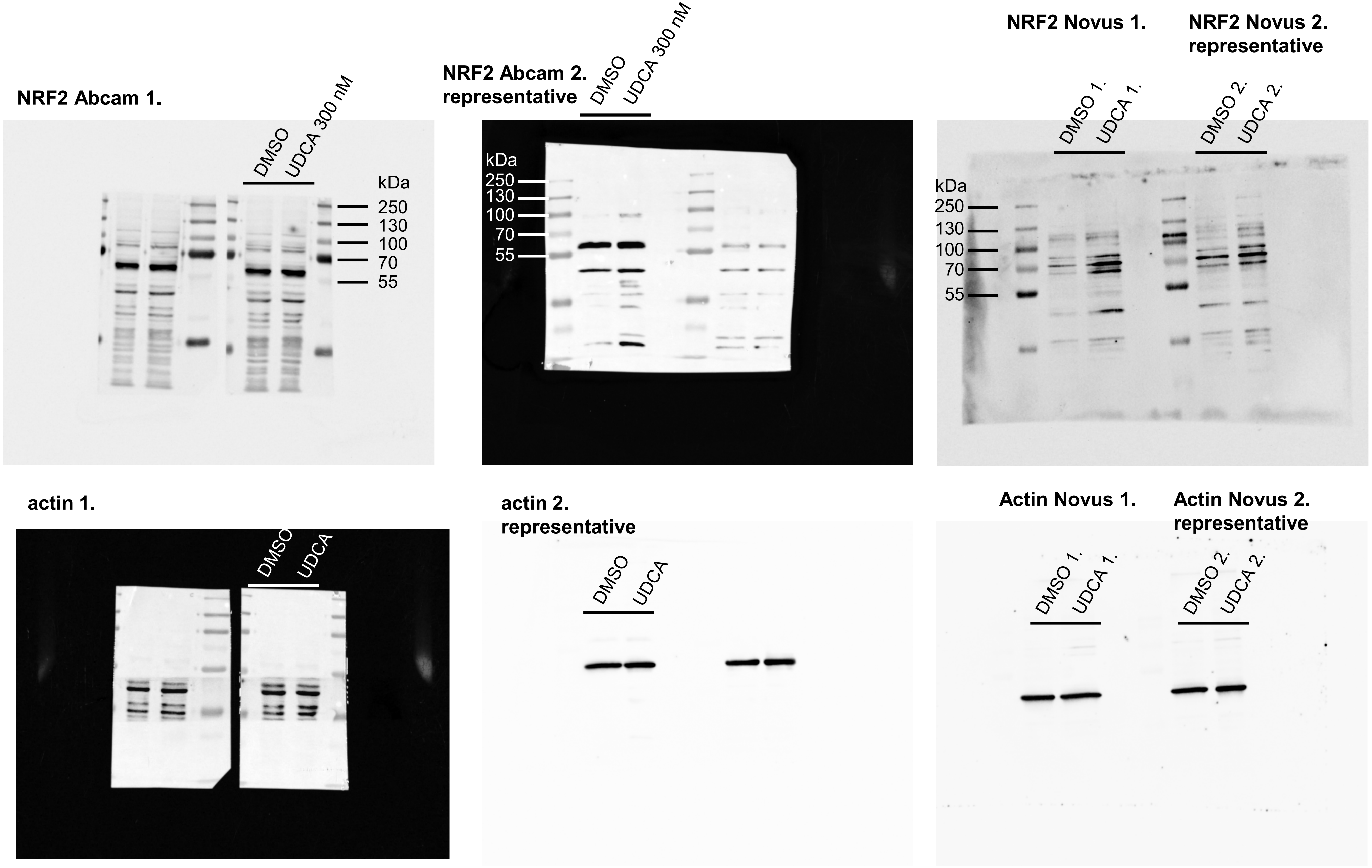

Figure 3. panel C: KEAP1

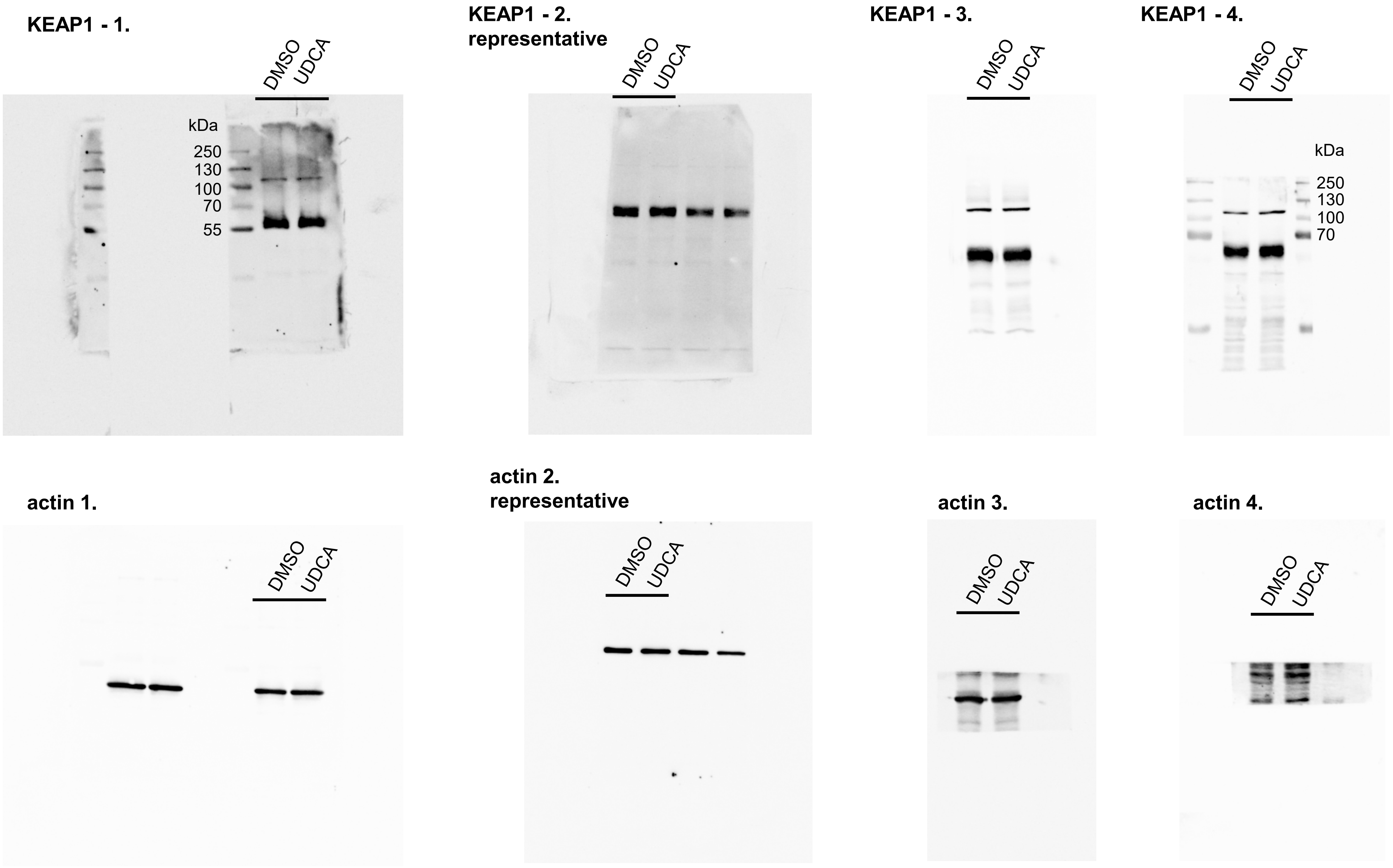

Figure 3. panel D: 4HNE

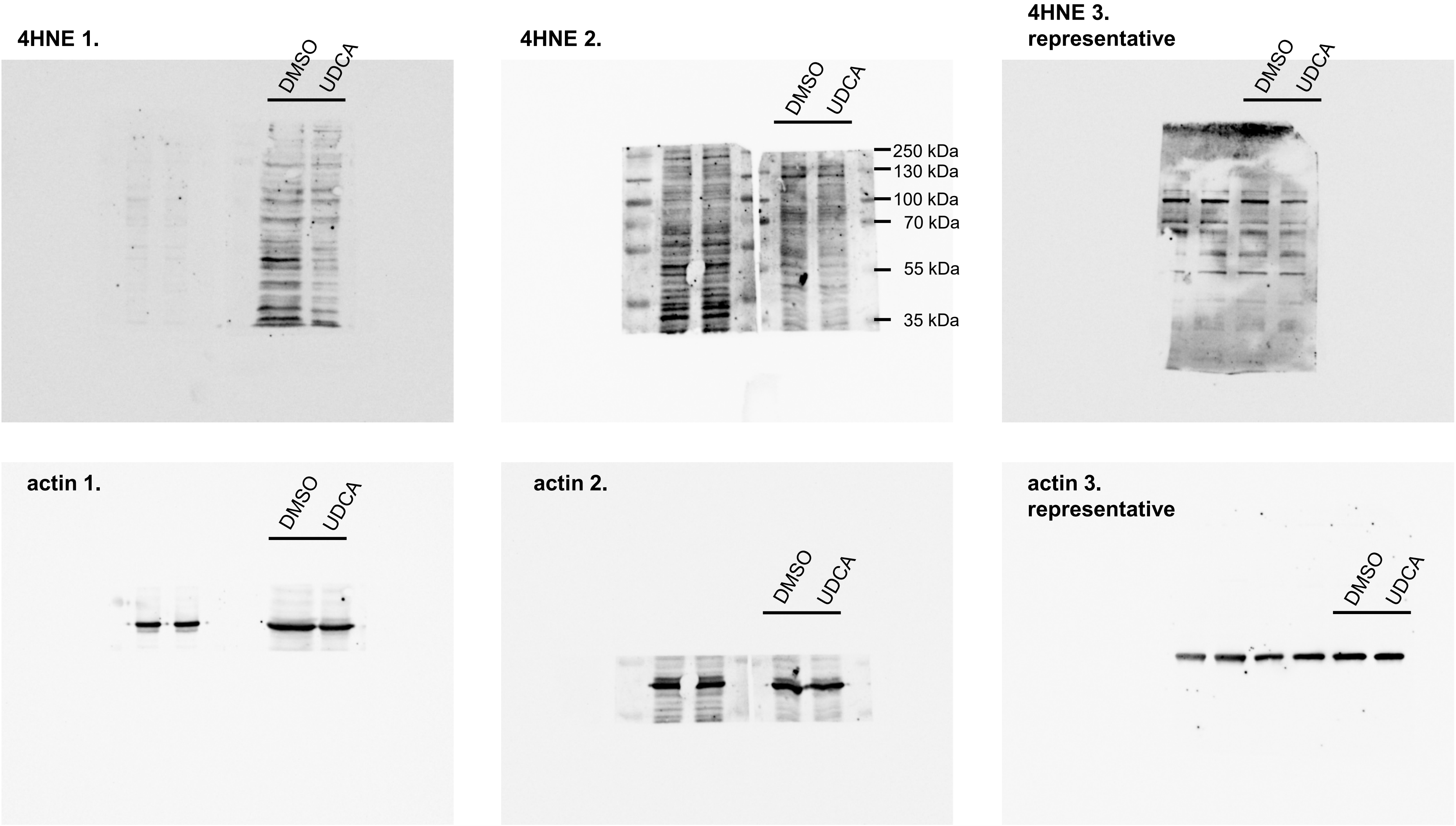

Figure 5. panel A: pS6K/S6K

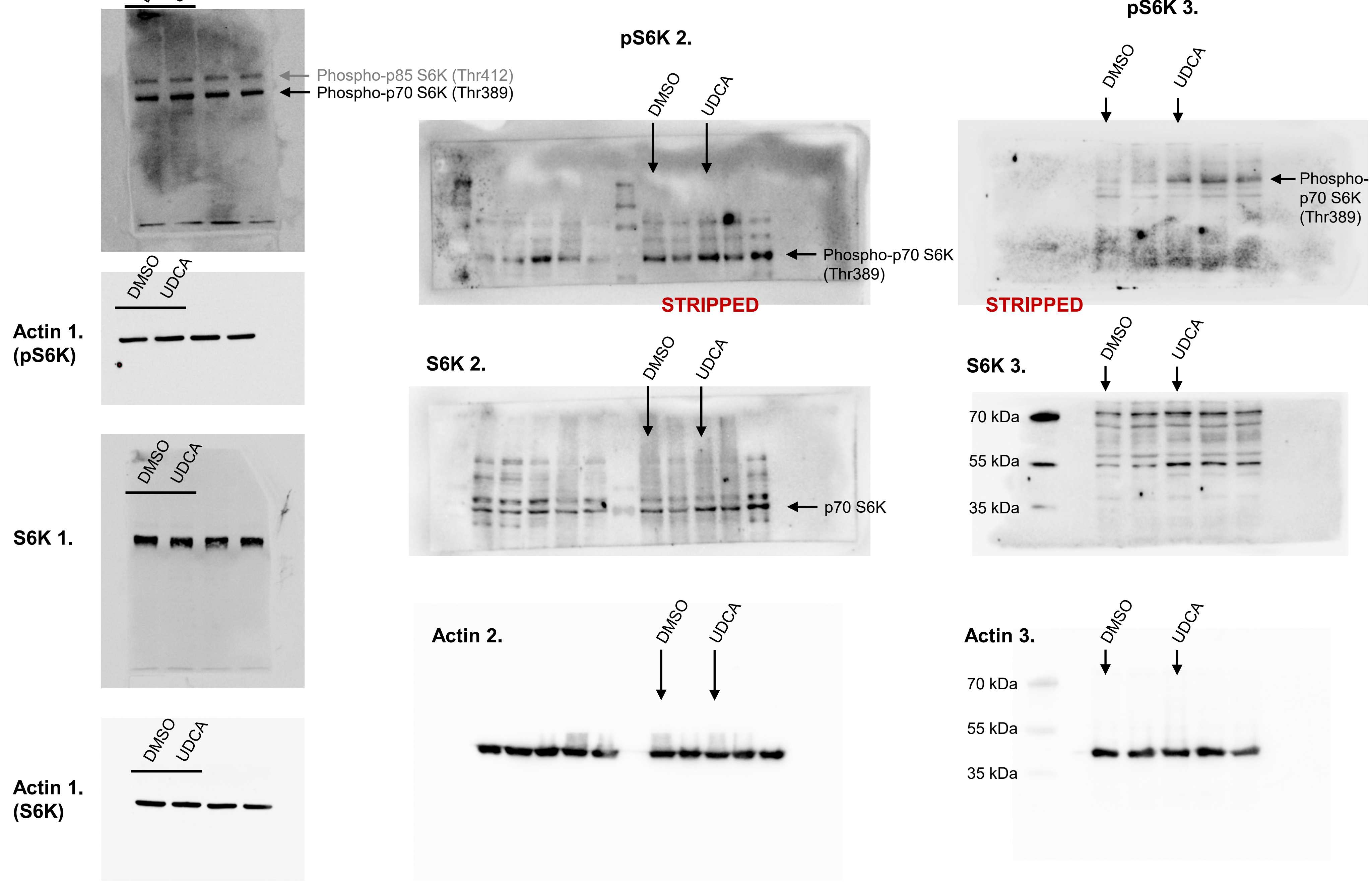

Figure 5. panel A: pS6K/S6K

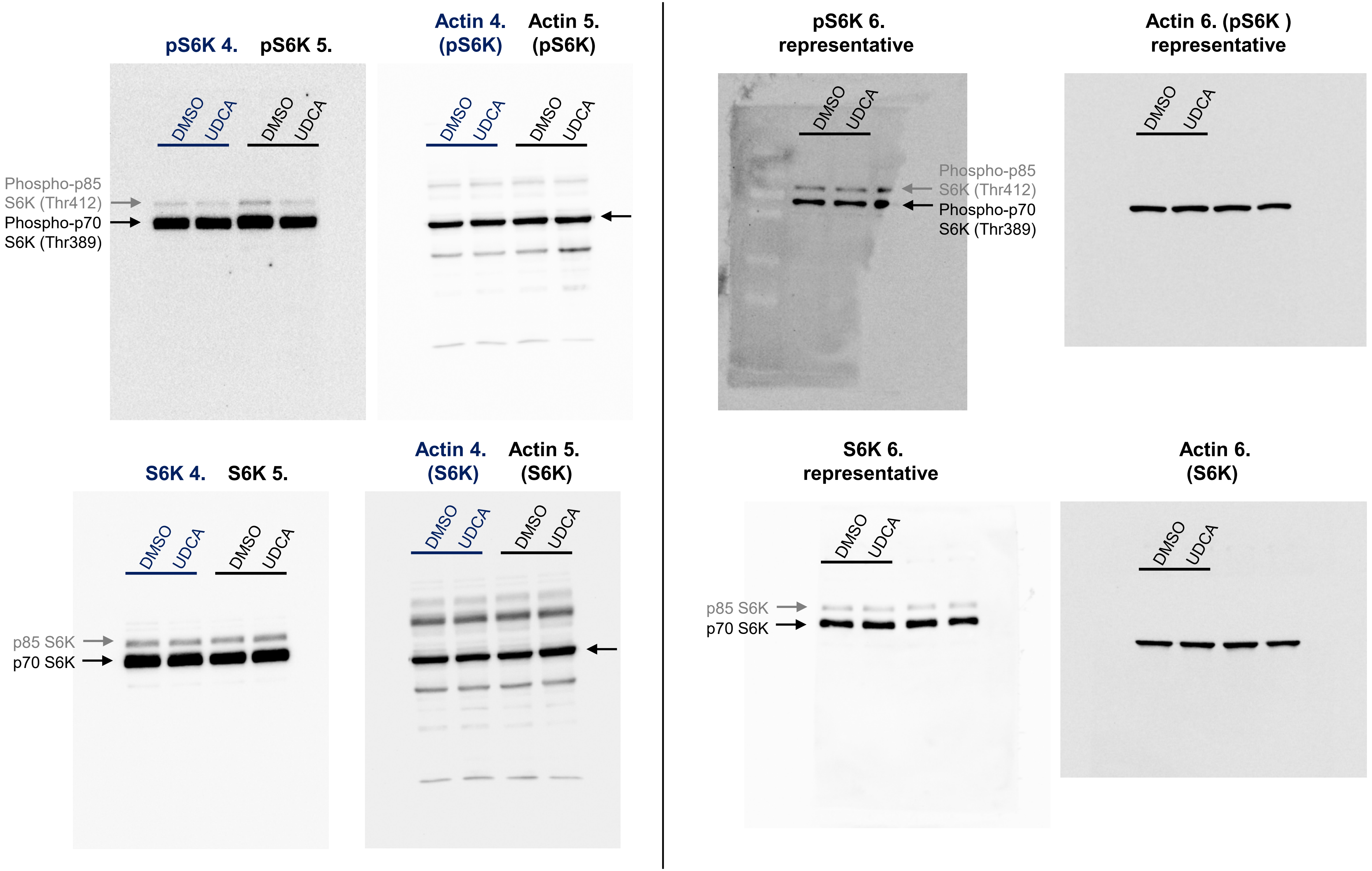

Figure 5. panel B: pAkt/Akt

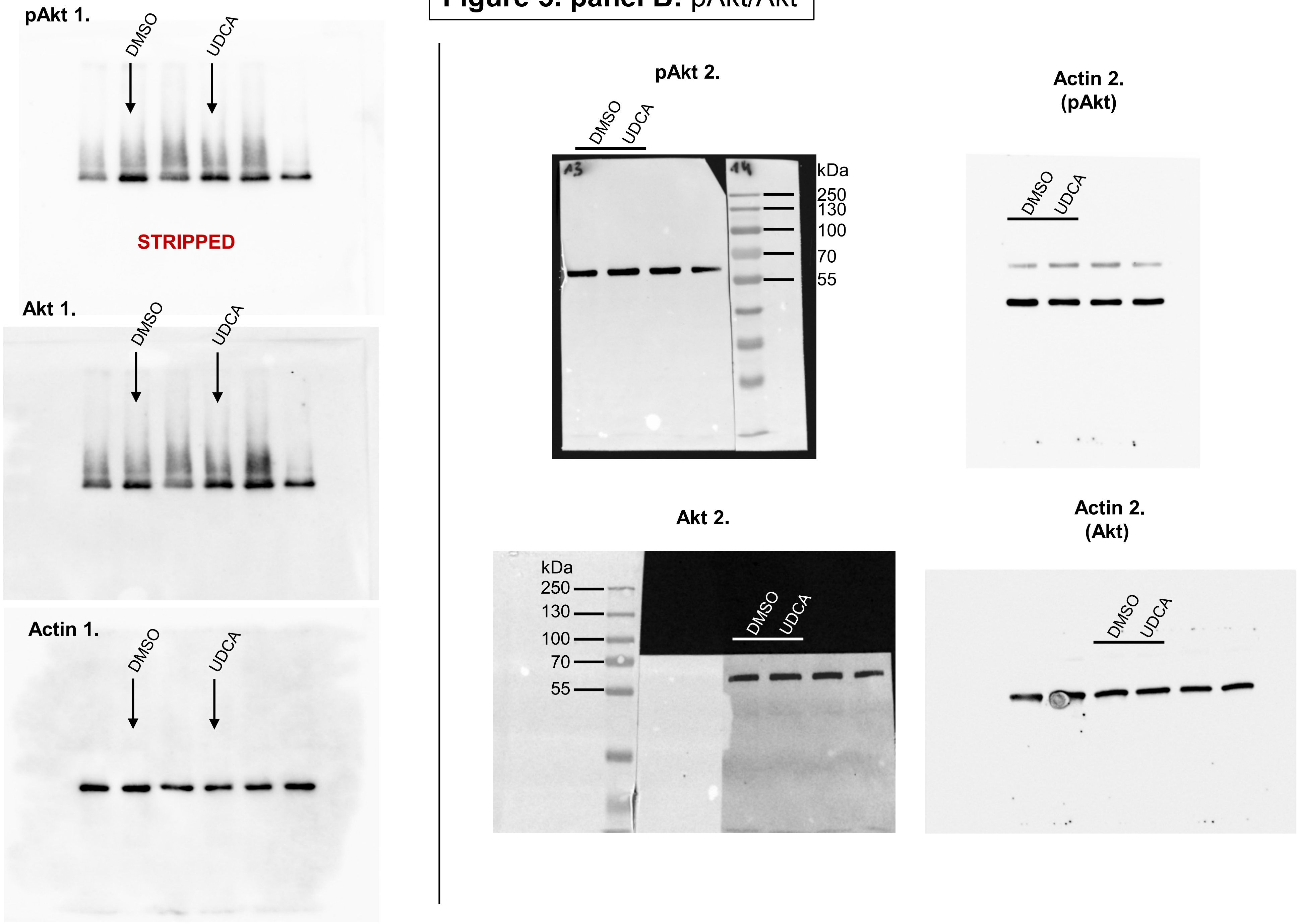

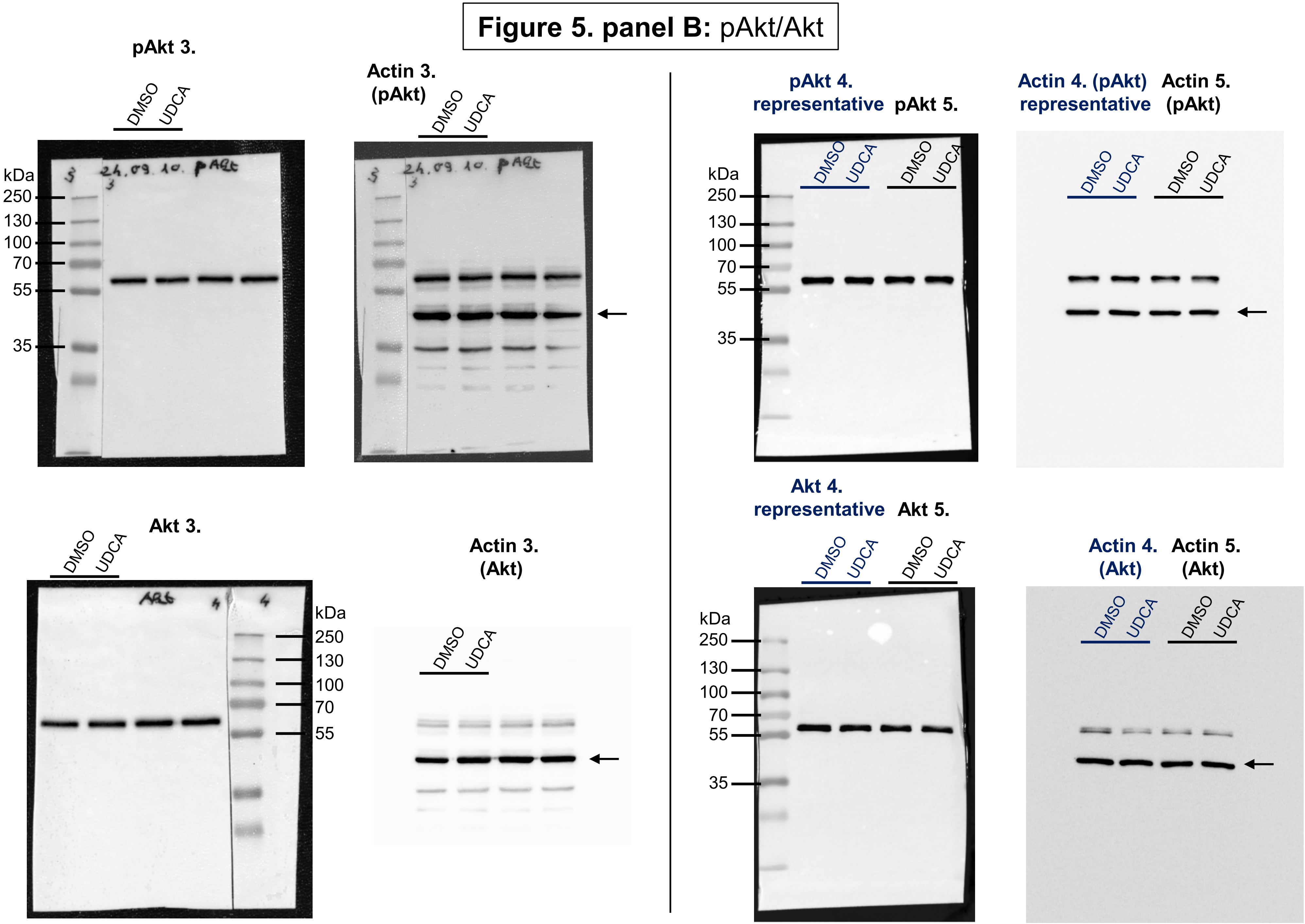
Figure 5. panel B: pAkt/Akt
pAkt 3.
DMSO
UDCA
kDa
250
130
100
70
55
35
24.09.10. pAkt
Actin 3.
(pAkt)
pAkt 4.
representative
pAkt 5.
Actin 4. (pAkt)
representative
Actin 5.
(pAkt)
Akt 3.
Akt
Actin 3.
(Akt)
Akt 4.
representative
Akt 5.
Actin 4.
(Akt)
Actin 5.
(Akt)

Figure 5. panel C: pAMPK/AMPK

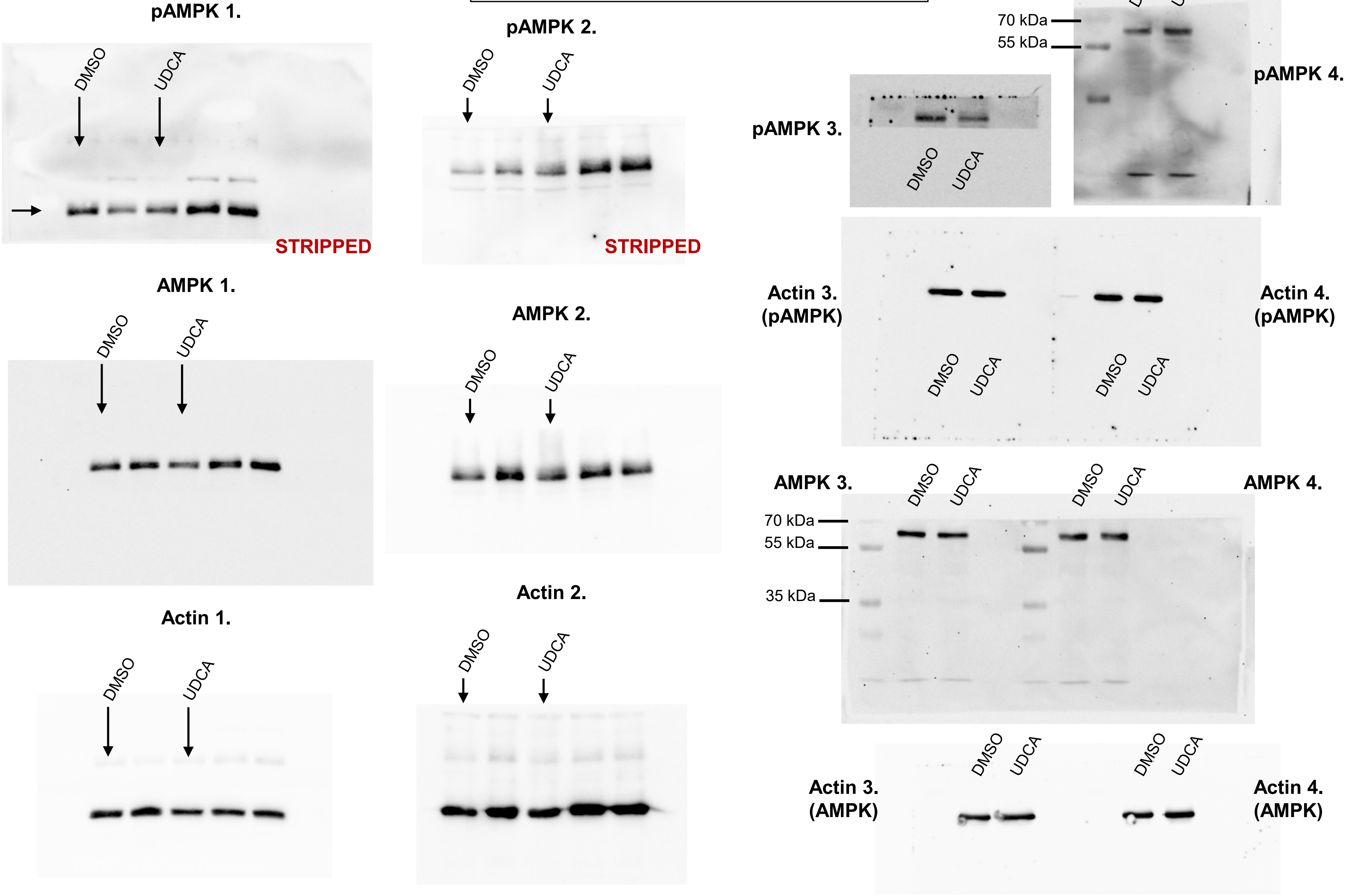

Supplement: Supplementary file 1 — Supplementary Material [file 41420_2025_2398_MOESM1_ESM.pdf]
